# Ex Vivo Human Placental Transfer of Rifampin and Rifabutin

**DOI:** 10.1155/S1064744996000646

**Published:** 1996

**Authors:** Kevin P. Magee, David Wimberley, Caren Crane, Sohrab Sobhi, Roger E. Bawdon

**Affiliations:** Department of Obstetrics and Gynecology University of Texas Southwestern Medical Center 5323 Harry Hines Boulevard Dallas TX 75235-9032 USA

## Abstract

*Objective:* The purpose of this study was to determine the ex vivo human placental transfer of rifampin and rifabutin.

*Methods:* Seven placentas from uncomplicated term vaginal or cesarean deliveries were studied utilizing the ex vivo single cotyledon perfusion system. Antipyrine was used for the reference compound in the determination of the clearance indices of rifampin and rifabutin.

*Results:* The clearance indices of rifampin at maternal concentrations of 1.0 and 10.0 μg/ml were 0.12 ± 0.05 and 0.12 ± 0.11, respectively. The clearance indices of rifabutin at maternal concentrations of 1.0 and 10.0 μg/ml were 0.44 ± 0.11 and 0.37 ± 0.15, respectively.

*Conclusions:* Because of its greater lipophilicity, rifabutin was found to have a greater clearance than rifampin. However, because of rifabutin's trend toward greater deposition in tissue, there was proportionately less accumulation of rifabutin in the fetal circulation when compared to rifampin.


Infectious Diseases in Obstetrics and Gynecology 4:319–322 (1996)
© 1997 Wiley-Liss, Inc.

### Ex Vivo Human Placental Transfer of Rifampin and Rifabutin

**Kevin P. Magee,* David Wimberley, Caren Crane, Sohrab Sobhi, and Roger E. Bawdon**

*Department of Obstetrics and Gynecology, University of Texas Southwestern Medical Center, Dallas, TX*

### Abstract

***Objective:*** **The purpose of this study was to determine the ex vivo human placental transfer of rifampin and rifabutin.**

***Methods:*** **Seven placentas from uncomplicated, term vaginal or cesarean deliveries were studied utilizing the ex vivo single cotyledon perfusion system. Antipyrine was used for the reference compound in the determination of the clearance indices of rifampin and rifabutin.**

***Results:*** **The clearance indices of rifampin at maternal concentrations of 1.0 and 10.0 µg/ml were 0.12 ± 0.05 and 0.12 ± 0.11, respectively. The clearance indices of rifabutin at maternal concentrations of 1.0 and 10.0 µg/ml were 0.44 ± 0.11 and 0.37 ± 0.15, respectively.**

***Conclusions:*** **Because of its greater lipophilicity, rifabutin was found to have a greater clearance than rifampin. However, because of rifabutin's trend toward greater deposition in tissue, there was proportionately less accumulation of rifabutin in the fetal circulation when compared to rifampin. Infect. Dis. Obstet. Gynecol. 4:319–322, 1996.** © 1997 Wiley-Liss, Inc.

Key words
maternal-fetal transfer; rifampin; rifabutin

Rifamycins are a class of antimicrobials that are macrocyclic products of *Streptomyces mediterranei*. The best known member of this class of antibiotics is rifampin, a derivative of rifamycin B, which is very effective in the treatment of *Mycobacterium tuberculosis* infections, when combined with isoniazid. Rifampin has a molecular weight of 822.95 g/mol and is lipophilic with a octyl alcohol to water (O/W) ratio of 16 at a pH of 7.4. It inhibits the DNA dependent RNA polymerase at the B ribosomal subunit. This inhibitory action is not noted in eukaryotic cells.[1,2]

Another member of this class of antibiotics is rifabutin, a semisynthetic derivative of rifamycin S, which is currently used in immunosuppressed individuals to provide prophylaxis against and treatment for *M. avium* complex (MAC) infections.[3,4] Rifabutin has also been shown to be effective in treating primary *M. tuberculosis* infections and cases of drug resistant *M. tuberculosis.*[5] Rifabutin's molecular weight is 847.02 g/mol and is also lipophilic with an O/W ratio of 2,562 at a pH of 7.0. Rifabutin's mechanism of action is similar to that of all rifamycins in that it also inhibits the microbe's DNA dependent RNA polymerase.[1,2]

Even though rifamycins were initially thought to be potentially teratogenic because of their ability to inhibit DNA dependent RNA polymerase, current evidence would indicate that they are safe for use in pregnancy. In one study of 446 pregnancies, the rate of malformations after exposure to rifampin was 3–4%, which is similar to that of the general obstetrical population and therefore is not thought to be significant.[6] There have been no

*Correspondence to: Dr. Kevin P. Magee, Department of Obstetrics and Gynecology, University of Texas Southwestern Medical Center, 5323 Harry Hines Boulevard, Dallas, TX 75235-9032.

Received 15 September 1996
Accepted 14 January 1997

studies of rifabutin use during human pregnancy. Rifabutin, therefore, has a category B rating by the Food and Drug Administration.

There is a paucity of studies in the literature regarding the transplacental passage of rifamycins. One study of rifampin use in early pregnancy analyzed the placental, fetal tissue, and amniotic fluid concentration of rifampin in a 13 week abortus. The patient had been receiving rifampin twice daily since the 2nd week of the gestation and received an oral dose of rifampin 4 h prior to the abortion. Rifampin was detected in all of the tissue analyzed.[7] Another study of the maternal-fetal transfer of rifamycins determined the rifampin concentrations in the neonate's blood.[8]

Rifampin's dosing during pregnancy typically follows the general guidelines of 10–20 mg/kg/day (given orally) up to a maximum daily dose of 600 mg/day.[9] Rifabutin, when used as prophylaxis against MAC, is given as a single daily dose of 300 mg orally.[3] Rifabutin prophylaxis is considered when patients with Centers for Disease Control (CDC) defined acquired immunodeficiency syndrome (AIDS) have a CD4 count of <200 cells/µl.

### MATERIALS AND METHODS

Rifampin and rifabutin were purchased and donated from Sigma Chemical Company (St. Louis, MO) and Adria Laboratories, Inc. (Dublin, OH), respectively.

The placentas utilized in this study were collected from term, uncomplicated, spontaneously delivered vaginal or cesarean delivered pregnancies in accordance with the guidelines set by the Institutional Review Board for Human Studies. The placentas were transferred from labor and delivery to the laboratory in normal saline in an aseptic manner. On arrival in the laboratory, the placenta's fetal artery and vein were cannulated with umbilical artery catheters. The total time from delivery to perfusion was approximately 7–10 min. The cannulated cotyledon was then gently perfused with Eagle's media and examined for evidence of loss of membrane integrity. Placental cotyledons that were noted to have fetal-to-maternal leaks were discarded. If there were no detectable leaks, the cotyledon and a portion of the placenta were transferred to a temperature controlled chamber and perfused with drug-free media for 20 min to remove any residual blood and stabilize the system. During this initial perfusion, the volume of the fetal perfusate was monitored closely for evidence of vascular leaks. The placental membrane integrity, clearance index, and transport fraction were determined by utilizing the $^{14}C$ antipyrine method of Challier.[10] A transfer fraction of >40% was deemed representative of a maternal-fetal circulatory match.

Both rifampin and rifabutin were studied separately and at two maternal concentrations (1.0 and 10.0 µg/ml) in both an open/open (non-recirculating) and a closed/closed (recirculating) system. Four placental perfusions were performed for the rifampin studies and three perfusions for the rifabutin studies. The maternal and fetal compartments consisted of 150 ml of medium, which was aerated via a frittered glass filter with a gaseous mixture of 95% air and $CO_2$. Both maternal and fetal compartments were continuously mixed by a magnetic stir bar. The fetal flow rate was set at 3–4 cc/min and the maternal flow rate was set at 17 cc/min.

Clearance indices of the two compounds were determined by perfusing a known concentration of the compound through the maternal circulation and collecting serial aliquots from the fetal circulation for analysis. During the determination of the clearance indices, the maternal and fetal circulations were open/open (i.e., non-recirculating).

The accumulation of rifampin and rifabutin in the fetal compartment was determined by placing a known concentration of the compound in the maternal circulation and perfusing the placenta in a recirculating manner (closed/closed). Again, serial aliquots were obtained every 10 min for 1 h to determine the accumulation of the antimicrobial. At the completion of the closed/closed perfusion of the 10 µg/ml experiment, the apparatus was carefully dismantled and an approximate 10 g sample of placental tissue was retrieved from the center of the blanched area of the perfused cotyledon for tissue concentration determination.

Concentrations of rifampin and rifabutin were determined by high performance liquid chromatography (HPLC) which utilized the 254 nM (ultraviolet) absorption spectrum for rifamycins and a standard C-18 bond-a-pack column (Waters Association, Milford, MA) and a mobile phase which was a modification of a published method for the isocratic determination of rifamycin-derived agents.[11] The modified mobile phase consisted of

TABLE 1. Clearance indices of rifampin and rifabutin from the perfusion studies utilizing an ex vivo human placenta with both the maternal and fetal circulations open (non-recirculating)[a]

| Drug | Concentration (μg/ml) | Clearance index | *P* |
|---|---|---|---|
| Rifampin (n = 4) | 1.0 | 0.12 ± 0.05 | |
| Rifabutin (n = 3) | 1.0 | 0.44 ± 0.11 | 0.01* |
| Rifampin (n = 4) | 10.0 | 0.12 ± 0.11 | |
| Rifabutin (n = 3) | 10.0 | 0.37 ± 0.15 | 0.05* |

[a]Each value represents the mean ± standard deviation of 6 samples over 1 h.
*Significant difference.

TABLE 2. Comparison of the accumulation of rifampin and rifabutin in the fetal compartment from perfusion studies utilizing an ex vivo human placenta with the maternal and fetal circulations closed (recirculating)[a]

| Drug | Concentration (μg/ml) | Fetal compartment concentration at 60 min (μg/ml) | *P* |
|---|---|---|---|
| Rifampin (n = 4) | 1.0 | 0.21 ± 0.09[b] | |
| Rifabutin (n = 3) | 1.0 | 0.18 ± 0.12[b] | 0.7* |
| Rifampin (n = 4) | 10.0 | 1.31 ± 1.78[b] | |
| Rifabutin (n = 3) | 10.0 | 0.86 ± 0.45[b] | 0.69* |

[a]Each value represents a total accumulation of 24 samples (n = 4).
[b]Standard deviation.
*Not significant.

50% acetonitrile for rifabutin and 40% acetonitrile for rifampin. These mobile phases were buffered by a 0.05 M $KH_2PO_4$ solution at a pH of 4.5. Perfusates and tissue samples were extracted with a one-to-one volume of 6% trichloroacetic acid. Reproducibility studies were determined for both rifampin and rifabutin.

Statistical analysis was performed by utilizing the t-test for the comparison of mean values between the two groups of data.

### RESULTS

Reproducibility studies for the HPLC determination of both rifampin and rifabutin concentrations in the perfusates and tissue samples were >95% with a minimum sensitivity of <0.1 μg/ml (data not shown).

Determinations of the clearance indices of rifampin and rifabutin revealed that rifabutin had a significantly higher clearance index than that of rifampin at both the 1.0 and 10.0 μg/ml concentrations (Table 1). There was no significant difference between rifampin's or rifabutin's accumulation in the fetal compartment (Table 2).

The determination of rifampin's and rifabutin's placental tissue concentrations noted a higher concentration of rifabutin in the tissue when compared to rifampin, but this was not statistically significant (Table 3). Rifabutin perfused placental tissue was noted to have 100% of the concentration of the maternal compartment. As expected, there was an appropriate decrease in the concentration of each drug in the maternal perfusate at the end of the closed/closed (recirculating) perfusion study (Table 4).

TABLE 3. Comparison of placental tissue concentrations of rifampin and rifabutin after 60 min of perfusion of a 10 μg/ml solution through an ex vivo human placental cotyledon with the circulations closed (recirculating)

| Drug | Placental tissue concentration (μg/g) | *P* |
|---|---|---|
| Rifampin (n = 4) | 3.37 ± 0.64[a] (27%) | |
| Rifabutin (n = 3) | 11.7 ± 8.7[a] (100%) | 0.105* |

[a]Standard deviation.
*Not significant.

TABLE 4. Initial concentration of rifampin and rifabutin in the maternal perfusate of an ex vivo human placenta

| Drug | Maternal compartment concentration (μg/ml) | Actual maternal concentration (μg/ml) |
|---|---|---|
| Rifampin (n = 4) | 1.0 | 0.86 ± 0.58[a] |
| | 10.0 | 12.4 ± 3.4[a] |
| Rifabutin (n = 3) | 1.0 | 1.17 ± 0.12[a] |
| | 10.0 | 11.7 ± 1.98[a] |

[a]Standard deviation.

### DISCUSSION

This comparison of rifampin and rifabutin in the ex vivo placental model noted a significant difference in the placental transfer of the two compounds. These differences can be explained by the difference in their lipophilicities. Rifabutin is several orders of magnitude more lipophilic than rifampin and therefore crosses the placenta more readily. Because of a large standard deviation in the analysis of the placental tissue concentration of rifabutin, the apparent difference did not reach statistical significance. However, because of its greater lipophilicity, rifabutin is more likely than rifampin to accumulate in the placental tissue. A comparison of tissue to plasma ratios from studies in non-pregnant patients reveals a greater ratio for rifabutin than rifampin. Specifically, these ratios were 1.05 to 0.83 for rifampin vs. 1.5 to 8.5 for rifabutin.[5,8] This apparent predilection for rifabutin to accumulate in tissues raises a concern about the drug's safety in pregnancy.

### REFERENCES

1. Hartman G, Honikel KO, Knusel F, Nuesch J: The specific inhibition of the DNA-directed RNA synthesis by rifamycin. Biochim Biophys Acta 145:843–844, 1967.
2. Umezawa H, Mizuno S, Yamazaki H, Nitta K: Inhibition of DNA dependent RNA synthesis by rifamycins. J Antibiot 21:234–236, 1968.
3. Nightingale SD, Cameron DW, Gordin FM, et al.: Two controlled trials of rifabutin prophylaxis against *Mycobacterium avium* complex infection in AIDS. N Engl J Med 329:828–833, 1993.
4. Summam PM, Gordin FM, Wynne BA, et al.: Efficacy of rifabutin in the treatment of disseminated infection due to *Mycobacterium avium* complex. Clin Infect Dis 19:84–86, 1994.
5. Brogden RN, Fitton A: Rifabutin: A review of its antimicrobial activity, pharmacokinetic properties and therapeutic efficacy. Drugs 47:983–1009, 1994.
6. Snider DE, Layde PM, Johnson MW, Lyle MA: Treatment of tuberculosis during pregnancy. Am Rev Respir Dis 122:65–79, 1980.
7. Rocker I: Rifampicin in early pregnancy. Lancet 1:48, 1977.
8. Kenny MT, Strates B: Metabolism and pharmacokinetics of the antibiotic rifampin. Drug Metab Rev 12:159–218, 1981.
9. American Thoracic Society, Centers for Disease Control: Treatment of tuberculosis and tuberculous infection in adults and children. Am Rev Respir Dis 134:355, 1986.
10. Challier JC: Criteria for evaluating perfusion experiments and presentation of results. Contrib Gynecol Obstet 13:32–39, 1983.
11. Moore DJ, Perrino PJ, Klerer CP, Robertson P: High-performance liquid chromatographic assay for two rifamycin-derived hypocholesterolemic agents in liver and biological fluids. J Chromatogr 612:310–314, 1993.
